# Effect of Phytase Level and Form on Broiler Performance, Tibia Characteristics, and Residual Fecal Phytate Phosphorus in Broilers from 1 to 21 Days of Age

**DOI:** 10.3390/ani12151952

**Published:** 2022-08-01

**Authors:** Jose R. Hernandez, Joseph P. Gulizia, John B. Adkins, Martha S. Rueda, Samuel I. Haruna, Wilmer J. Pacheco, Kevin M. Downs

**Affiliations:** 1 Department of Poultry Science, Auburn University, Auburn, AL 36849, USA; jzh0237@auburn.edu (J.R.H.); jzg0120@auburn.edu (J.P.G.); msr0036@auburn.edu (M.S.R.); wjp0010@auburn.edu (W.J.P.); 2 School of Agriculture, Middle Tennessee State University, Murfreesboro, TN 37132, USA; jba0030@auburn.edu (J.B.A.); samuel.haruna@mtsu.edu (S.I.H.)

**Keywords:** phytase, broilers, growth performance, bone mineralization, phytate phosphorus

## Abstract

**Simple Summary:**

The demand for poultry products is increasing at the same time as the prices of raw materials and other ingredients used in feed manufacturing, leading to the implementation of strategies to improve nutrient utilization in broiler diets and reduce feeding costs. One strategy used in broiler production is the dietary supplementation of phytase, an enzyme that assists in phytate degradation. Phytate is the primary storage form of phosphorus (P) in seeds and it accounts for two-thirds of the P in vegetable feedstuffs. Dietary phytase supplementation has become a common practice because the activity of endogenous phytase in broilers does not allow for the adequate utilization of phytate-bound P. Consequently, the effects of phytase have been widely studied and the beneficial effects on broiler growth performance and reduced P excretion into the environment have been previously described. In the present study, broilers were fed a diet with a combination of two phytase forms (coated and uncoated) to assess the effects on broiler performance, tibia characteristics, and residual fecal phytate P. The results indicated no distinct advantage of combining phytase forms, but this information may be useful for the direction of future research.

**Abstract:**

The present study evaluated the individual and combined effects of coated and uncoated phytase on broiler performance, tibia characteristics, and residual phytate phosphorus (P) in manure. Two repeated studies were conducted using 240-day-old Cobb 500 by-product male broilers per trial. For each trial, birds were assigned to four treatments with four replicate battery cages per treatment (60 birds/trt) and grown for 21 days. Treatments included: (1) negative control (NC), (2) NC + 1000 phytase units (FTU) coated phytase (C), (3) NC + 1000 FTU uncoated phytase (U), and (4) NC + 500 FTU coated + 500 FTU uncoated phytase (CU). Data were analyzed with a one-way ANOVA and means were separated using Tukey’s HSD. In the pooled data for both trials, all treatments with dietary phytase had a higher body weight (BW) and feed consumption (FC) than the NC on day 21 (*p* < 0.05). Similarly, a six-point reduction was observed for day 1 to 21 feed conversion (FCR) for U and CU (*p* < 0.05). All treatments with phytase inclusion differed from the NC in every evaluated parameter for bone mineralization (*p* < 0.05) and had significantly lower fecal phytate P concentrations compared to the NC (*p* < 0.05). Overall, bird performance was essentially unaffected by phytase form, indicating that combining phytase forms does not appear to offer any advantage to the evaluated parameters from day 1 to 21.

## 1. Introduction

The increasing demand for poultry meat and higher feed prices have led to the implementation of nutritional strategies that optimize nutrient utilization and reduce feeding costs. Among these strategies is the dietary inclusion of phytase, which reduces the nutrient variability of feedstuffs and counteracts the antinutritional effects of phytate [1]. Phytase inclusion has become a common practice in broiler feeding as a means of increasing the hydrolysis of phytate, improving phytate phosphorus (P) utilization, and reducing P excretion [2].

Phytate (myo-inositol (1,2,3,4,5,6) hexakisphosphate; IP_6_) is the salt form of phytic acid (PA) and the primary storage form, typically contributing 50 to 80% of total P, in plant seeds [3]. Chemically, it consists of a myo-inositol ring associated with up to six phosphate anions [4]. Since broiler diets are mainly manufactured with feedstuffs of plant origin, the antinutritive effects of phytate in broiler production have been extensively reviewed [5,6]. Approximately two-thirds of P in vegetable feedstuffs is poorly digested by poultry because it is bound to PA; birds do not produce sufficient amounts of endogenous phytase to effectively hydrolyze PA [7]. Additionally, since phytate is negatively charged under many pH conditions, it can form complexes with positively charged molecules, which may reduce the bioavailability of minerals, amino acids, and protein [8,9,10]. There are reports that phytate also reduces endogenous enzyme activity in broilers [11]. Ultimately, phytate can negatively affect bird performance and increase the amount of P excreted into the environment.

Phytases (myo-inositol (1,2,3,4,5,6) hexakisphosphate phosphohydrolases) represent a subgroup of phosphatases that are capable of initiating phytate dephosphorylation, thus releasing phosphate groups from PA [7,9]. These enzymes are often sourced from bacteria and fungi and can be classified as 3-phytases or 6-phytases depending on the carbon site of hydrolysis in the phytate molecule [12]. Phosphate group removal starts with a fully phosphorylated PA, followed by penta-, tetra-, tri-, di- and mono-esters of inositol in descending order of preference [5,13,14]. In addition, the release of inorganic P depends on several factors, including, but not limited to, dietary phytate concentration, source of phytate, species and age of animals, mineral concentrations in the diet, phytase sources, and phytase dosing [15].

Inorganic P sources are expensive and represent the third most expensive component of poultry diets after energy and amino acids [7]. Furthermore, P excretion into the environment is a hazard to water quality because P is a predisposing factor for the eutrophication of freshwater bodies [16]. Therefore, the use of phytase is beneficial economically and for sustainable production. Previous studies have determined that the inclusion of phytase improves broiler performance by increasing feed consumption (FC) and body weight (BW) and decreasing feed conversion ratio (FCR) [17,18,19,20,21,22]. Similarly, phytase supplementation has proven beneficial to bone mineralization from improved P digestibility [23,24].

Commercially available phytases may differ in several characteristics, including form and origin. Some phytases are encapsulated by lipid or carbohydrate coating to minimize oxidation at high temperatures, particularly during pelleting conditions [25]. Thermostability is an important characteristic of phytases, as animal diets are frequently pelleted at temperatures up to 90 °C [26], but evaluating this attribute is not within the scope of this study. Previous studies have assessed the activity of coated and uncoated phytases on broiler performance and tibia mineralization. Ayres et al. [12] evaluated the activity of a coated phytase and an uncoated phytase developed to be intrinsically heat-stable on 21-day broiler performance and tibia mineralization. They concluded that the uncoated and intrinsically heat-stable phytase was more efficacious than coated phytase. However, there is limited information regarding the effect of combining different phytase forms (coated and uncoated) in the same diet. Therefore, the objective of this study was to determine the effects associated with combining coated and uncoated phytase sources on broiler performance, tibia characteristics, and residual phytate P in broiler manure from 1 to 21 days of age (starter phase).

## 2. Materials and Methods

### 2.1. Diet Preparation

Two repeated trials were conducted to fully elucidate the effect of phytase form and inclusion on broiler performance, tibia characteristics, and residual fecal phytate P. Coated (OptiPhos^®^; Huvepharma Inc., Peachtree City, GA, USA) and uncoated (OptiPhos^®^ Plus; Huvepharma Inc., Peachtree City, GA, USA) phytase was incorporated into a basal broiler starter diet (mash form) as either a single inclusion or a combination to create four experimental diets (Table 1). Both products are 6-phytase derived from *E. coli*. The basal diet was manufactured as a negative control (NC) with reduced calcium (Ca) and available P (aP) levels; however, all remaining nutrients represented a typical broiler starter diet. The NC diet was formulated with 0.20% less Ca and aP compared to industry standards [27] and National Research Council (NRC) requirements [28]. Three additional treatments were manufactured using the NC with different combinations and forms of phytase products. Two treatments contained either a single inclusion of a coated (1000 FTU/kg complete feed; FTU = phytase units) or uncoated (1000 FTU/kg complete feed) phytase, whereas one treatment contained a combination of coated (500 FTU/kg complete feed) and uncoated (500 FTU/kg complete feed) phytase. The addition of 1000 FTU of phytase activity per kg of complete feed was expected to increase Ca and P availability by 0.20%.

Dietary treatments were prepared at the Auburn University Animal Nutrition Center as described in Downs et al. [29]. The phytase supplement was premixed with ground corn prior to its addition to the whole feed batch. After mixing, coated, uncoated, or the combination of both phytase products were added to the basal diet and mixed for 5 min to prepare each treatment with dietary phytase inclusion. The starter feed used in each treatment met or exceeded Cobb 500 nutrient recommendations [27] and NRC requirements [28], except for Ca and aP.

### 2.2. Phytase Analysis

Feed samples were collected during feed manufacture and analyzed for phytase activity levels by an external laboratory. The analyzed phytase activity for each treatment is shown in Table 2.

### 2.3. Bird Management and Data Collection

Animal handling procedures were approved by the Middle Tennessee State University Institutional Animal Care and Use Committee (IACUC) under proposal 21–2001 and conformed to accepted animal welfare standards [30,31]. Both trials were conducted using 240-day-old Cobb 500 by-product males obtained from a local hatchery. Chicks were randomly allocated to 16 battery cages (15 birds per cage; 527 cm^2^/bird) and each cage was randomly assigned to a treatment (4 treatments; 4 replicate cages/treatment; 60 birds/treatment). Birds were brooded at 35 °C with the temperature reduced by 3 °C every 7 days. The temperature of the room housing battery cages was maintained at 27 °C and continuous light was provided (24 L:0 D; 25 lux), with feed and water offered *ad libitum.* Bird spacing for feeders (9.6 cm/bird) and drinkers (4.8 cm/bird) met or exceeded the recommendations for broilers grown to 21 days of age [30,31].

All birds and feed were weighed at days 1, 14, and 21 to determine average BW and FC. Feed conversion was calculated using FC and BW gain data and adjusted for mortality. Mortality weights were included in the FCR calculation to determine mortality-adjusted values.

On day 21, left tibias were collected to assess shear strength and ash content. For this procedure, 5 birds per cage were randomly selected and euthanized according to AVMA guidelines [32]. Tibias were evaluated for shear strength using a TA.XT plus texture analyzer (Stable Microsystems, Surrey, UK) according to the official method (ANSI/ASAE method s459) [33]. A test speed of 5 mm/sec and a trigger force of 5 g were used. Applied fracture force was recorded in newtons (N) and determined as the peak force at the initial fracture. Peak 1 was considered the force to crack the bone and peak 2 was considered the force to break the bone. After breaking tibias and analyzing for shear strength, each bone was exposed to sequential extractions using 200-proof ethanol and anhydrous ether and ashed in a muffle furnace, as described by Hall et al. [34].

Excreta samples were collected on day 21 to assess phytate P level. An aliquot of fresh excreta was collected from the refuge tray of each battery cage, screened to remove non-fecal material, and frozen (−4 °C) for further analysis. Excreta samples were analyzed at the University of Georgia Feed and Environmental Water Laboratory (Agricultural and Environmental Services Laboratories, Athens, GA, USA) for phytate P level determination using a standard extraction-colorimetric procedure.

### 2.4. Statistical Analysis

Data were analyzed as a completely randomized block design, with a battery cage representing an experimental unit and trials treated as replicates. Data presented were pooled from both trials. Mortality data were arcsine-transformed. Data were analyzed using the general linear model (GLM) procedure of the SAS statistical package [35]. Least square means were compared using post hoc Tukey’s HSD procedure, and all data were analyzed for normality using the Shapiro–Wilk test. Statistical significance was established at *p* ≤ 0.05. Statistical tendency was considered as 0.05 < *p* < 0.10.

## 3. Results

Phytase activity was determined in the four dietary treatments (Table 2). Diets without phytase inclusion had less than 13 FTU/kg. The treatments with phytase supplementation had an expected value of 1000 FTU/kg and the analyzed value had a slight variation between treatments. Phytase recoveries were 118, 109, and 115% for NC + Coated (C), NC + Uncoated (U), and NC + Combined (CU), respectively.

Performance results (Table 3) and other evaluated parameters were pooled from trials 1 and 2. For day 14 BW, CU was the only treatment not different from NC (*p* > 0.05). All phytase-treated birds had higher day 21 BW than NC (*p* < 0.05) regardless of the phytase form used. Feed consumption was the only parameter that did not differ across treatments from day 1 to 14 (*p* > 0.05), but from day 1 to 21, all three treatments containing phytase presented a higher FC than the NC (*p* < 0.05). In accordance with the results for BW, the data for FCR suggest that the use of phytase reduced FCR between 4 and 6 points. From day 1 to 14, C and CU both reduced FCR by 5 points compared to the NC (*p* < 0.05). Similarly, a 6-point reduction was observed for day 1 to 21 FCR for U and CU (*p* < 0.05). However, days 1 to 14 FCR of U did not differ from the NC and the same was observed for the C treatment from 1 to 21 days of age (*p* > 0.05). In addition, the pooled data indicate that treatments did not have an effect on bird mortality (*p* > 0.05).

Tibias were assessed for bone weight, shear strength, and tibia ash percentage (Table 4). The obtained results indicate that all treatments with phytase inclusion differed from the NC in every bone parameter evaluated (*p* < 0.05). Tibia ash increased around 5% in diets with phytase inclusion and bone weight increase was 8, 10, and 12% for C, U, and CU, respectively (*p* < 0.05).

For fecal phytate P, significant differences were observed across treatments in the pooled data for both trials (Table 5). All treatments with dietary phytase inclusion had significantly lower fecal phytate P concentration compared to the NC (*p* < 0.05). The reduction in phytate P concentrations in feces were 74, 79, and 77% for C, U, and CU, respectively. In addition, no differences in fecal phytate P concentrations were observed between the phytase-containing treatments (*p* > 0.05).

## 4. Discussion

Birds poorly utilize phytate-bound P due to their limited endogenous phytase production. Previous studies have reported that the inclusion of exogenous phytase can reduce the negative effects of phytate on nutrient utilization, resulting in phytase products being commonly utilized in poultry diets. As mentioned previously, coated and uncoated phytases have been evaluated to determine their effects on broiler performance, bone mineralization, and phytate hydrolysis. However, there is limited information on the effects of combining phytase forms. In this regard, it is important to mention that bird age can influence the utilization of nutrients and the efficacy of phytase in broiler chickens [36,37]. Broilers are able to utilize P more efficiently and phytase is more efficacious during the starter phase, with a higher sensitivity during the end of the second week of the growing period [37]. This period of rapid growth and development serves as a foundation for the deposition of meat in the subsequent phases [38], hence the importance of reporting results from the starter phase.

The BW results of this study were similar to the data obtained by Leyva-Jimenez et al. [39]. The authors reported that broilers fed diets supplemented with regular (250 FTU/kg) and superdose (1500 FTU/kg) levels of Optiphos 2000 (Huvepharma Inc. Peachtree, GA, USA) had a significantly higher day-22 BW in comparison to birds fed no phytase. Ayres et al. [12] used the same two products evaluated in the present study with increasing inclusion levels. In two of their experiments, they observed higher day-21 BW for birds fed diets with 1000 FTU of an uncoated phytase, compared to birds fed diets with the same inclusion of a coated phytase and a negative control with no phytase inclusion. Several studies have obtained similar results of increased body weight and feed intake with phytase supplementation at low and high inclusion levels [40,41]. This may be caused by the activity of phytase in the gastrointestinal tract, which contributes to the breakdown of IP_6_ and lesser phytate esters, therefore increasing the availability of phytate-bound nutrients [41,42]. Additionally, these data indicate that apart from improving nutrient utilization, phytase also stimulates a feed intake response and, consequently, improves BW. It has been suggested that the effects of phytase in stimulating digestible nutrient intake may be associated with its impact on phytate degradation since phytate reduces feed intake in broilers [43,44].

The results for FCR that were obtained in this study suggest that phytase inclusion in diets with reduced Ca and aP is able to prevent the P deficiency effects on growth performance parameters, thus allowing birds to match the performance of birds fed nutrient-adequate diets [41]. Dersjant-Li et al. [45] obtained similar results when using a dose of 1000 FTU/kg of a coated phytase in diets with different reduced Ca levels. The authors reported a lower day 11 to 21 FCR in broilers fed phytase-supplemented diets, compared to an NC with no phytase inclusion and no Ca reduction. Similarly, Broch et al. [46] evaluated increasing levels of phytase inclusion (1000, 2000, and 3000 FTU/kg) in broiler diets deficient in aP and Ca and observed a linear response for all growth performance parameters from 1 to 21 days of age. In the present study, combining two different phytase forms did not appear to offer any distinct performance advantages. The benefits of combining different phytase forms could have been observed if they were released at different rates throughout the gastrointestinal tract of broilers. Exogenous phytase is, for the most part, active in the proximal segments of the gastrointestinal tract (crop, proventriculus, and gizzard) of poultry [47]. If combining phytase forms could extend phytase activity to the upper part of the small intestine, it would reduce the binding of phytate to dietary protein and the formation of calcium phytate complexes, thus reducing its antinutritional effects and endogenous amino acid losses [3,10]. Nevertheless, the results obtained in this study do not indicate any clear benefits of combining a coated and uncoated phytase product in the growth performance of broilers.

According to Cardoso Junior et al. [48], bone mineralization reflects adequate bone quality, which is associated with beneficial effects on broiler performance and is important to support muscular development. Furthermore, bone mineralization data is commonly used to estimate and validate inorganic P release by phytase and is an efficient parameter to quantify phytate P released in corn and soybean meal-based diets [3,49]. It is worth noting that Ca and P are closely related in bone mineralization results because they are both stored together in bone and Ca is stored almost entirely as hydroxyapatite crystals of Ca phosphate in bone [50]. In this study, the three treatments with phytase supplementation had improved results for all bone parameters in comparison to the NC. Similarly, Chung et al. [51] reported improvements in bone mineral content and bone mineral density in broilers fed diets with two inclusion levels (500 and 1000 FTU/kg) of a coated phytase compared to broilers fed low-P diets. In addition, Walk et al. [20] and several other authors have observed that tibia ash and other bone parameters improve with phytase addition [36,39,52]. This indicates a positive effect of phytase in bone mineralization, which is associated with an increase in the amount of aP [41]. Moreover, these results suggest that phytase supplementation is beneficial for bone mineralization and is in accordance with the data obtained for growth performance measurements. However, no clear effects were observed for the combination of different phytase forms.

The results for phytate P concentrations in feces from 21-day-old broilers demonstrate the beneficial effect of phytase on phytate degradation. The feces from broilers fed phytase-supplemented diets had reduced fecal phytate P concentrations compared to the NC and no differences were observed between treatments combining phytase forms. Martins et al. [53] observed a reduction in residual fecal phytate P of broilers fed phytase-supplemented diets (750 FTU/kg) compared to broilers fed diets with no phytase addition. Kriseldi et al. [54] reported similar results when evaluating two doses of phytase (400 and 1200 FTU/kg) on phytate concentration in the ileal digesta of 28-day-old birds. The authors reported that both concentrations of phytase effectively increased phytate degradation and this led to increased inositol liberation. It is essential to achieve the maximum degradation of phytate to obtain the extra-phosphoric effects of phytase and observe improvements in the growth performance of broilers [55]. In addition, the degradation of IP_3_, IP_4_, and lower esters is critical, as the anti-nutritive effects of these lower IP esters may still be present to chelate nutrients [56]. Therefore, more research is required to assess the effect of combining different phytase forms in the degradation of IP esters and the liberation of inositol. Furthermore, reductions in the P excretion of broilers can reduce the environmental pollution burden, since P from poultry manure can pollute soil and is a significant eutrophication agent [57].

## 5. Conclusions

The results obtained in this study are consistent with previous research on phytase and its effects on broiler performance, tibia characteristics, and residual fecal phytate P. Improvements in BW, FC, and FCR were observed when using 1000 FTU/kg of dietary phytase, regardless of its form. Phytase addition also improved tibia characteristics and reduced residual phytate P in broiler feces. However, the combination of phytase forms does not appear to offer any distinct advantage to bird performance or bone mineralization from day 1 to 21. Although the phytase research area has been widely studied, a paucity of research exists that evaluates the effects of combining two different phytase forms (coated and uncoated) in the same broiler diet. Therefore, further research is needed to better understand if combining different phytase forms represents potential benefits for broiler growth.

## Figures and Tables

**Table 1:** Ingredient and analyzed nutrient compositions of the negative control (NC) diet (as-fed basis).

**Ingredient, % of Diet (Unless Otherwise Noted)**	**NC**
Corn	51.60
Soybean meal, 46% crude protein	37.94
Corn oil	3.31
Distillers dried grains with solubles	4.00
Dicalcium phosphate	0.55
Ground limestone	1.45
Salt (NaCl)	0.38
DL-Methionine	0.33
L-Lysine	0.18
Trace mineral premix ^A^	0.10
Vitamin premix ^B^	0.10
Choline chloride	0.07
Phytase, g supplement/kg diet	0.00 ^C^
**Calculated Nutrients, % (Unless Otherwise Noted)**	
AMEn, kcal/kg	3000
Crude protein	23.17
Calcium	0.80
Available phosphorus	0.20
Digestible methionine	0.64
Digestible methionine + cysteine	0.93
Digestible lysine	1.23
Digestible threonine	0.73
Digestible valine	0.96
Digestible tryptophan	0.25

^A^ Trace Mineral premix source and amount provided per kg of diet: Mn (manganese sulfate), 120 mg; Zn (zinc sulfate), 100 mg; Fe (iron sulfate monohydrate), 30 mg; Cu (tri-basic copper chloride), 8 mg; I (ethylenediamine dihydriodide), 1.4 mg; and Se (sodium selenite), 0.3 mg. ^B^ Vitamin premix source and amount provided per kg of diet: Vitamin A (Vitamin A acetate), 18,739 IU; Vitamin D (cholecalciferol), 6614 IU; Vitamin E (DL-alpha tocopherol acetate), 66 IU; menadione (menadione sodium bisulfate complex), 4 mg; Vitamin B12 (cyanocobalamin), 0.03 mg; folacin (folic acid), 2.6 mg; D-pantothenic acid (calcium pantothenate), 31 mg; riboflavin (riboflavin), 22 mg; niacin (niacinamide), 88 mg; thiamine (thiamine mononitrate), 5.5 mg; D-biotin (biotin), 0.18 mg; and pyridoxine (pyridoxine hydrochloride), 7.7 mg. ^C^ Using the NC, 3 treatments were supplemented with either OptiPhos^®^ (0.126 g/kg; Huvepharma Inc., Peachtree City, GA, USA), OptiPhos^®^ Plus (0.090 g/kg; Huvepharma Inc., Peachtree City, GA, USA), or a combination of both phytase products (0.108 g/kg) to achieve 1000 FTU of phytase activity per kg of diet.

**Table 2:** Analysis of phytase activity in the negative control (NC) and phytase-supplemented diets.

Item	NC	NC + Coated ^A^	NC + Uncoated ^B^	NC + Combined ^C^
Expected value, FTU/kg	0	1000	1000	1000
Analyzed value, FTU/kg ^D^	<13	1180	1090	1150

^A^ OptiPhos^®^ (Huvepharma Inc., Peachtree City, GA, USA) added to provide 1000 FTU/kg of phytase activity per kg of diet. ^B^ OptiPhos^®^ Plus (Huvepharma Inc., Peachtree City, GA, USA) added to provide 1000 FTU/kg of phytase activity per kg of diet. ^C^ OptiPhos^®^ and OptiPhos^®^ Plus combined to provide 1000 FTU/kg of phytase activity per kg of diet. ^D^ Phytase was analyzed by an external laboratory (Huvepharma; BIOVET laboratory for feed analysis, Peshtera, Bulgaria).

**Table 3:** Influence of negative control (NC), coated phytase supplementation (NC + 1000 FTU ^A^ coated phytase), uncoated phytase supplementation (NC + 1000 FTU uncoated phytase), and combined phytase supplementation (NC + 500 FTU coated + 500 FTU uncoated) on broiler live performance from 1 to 21 days of age (pooled data from trial 1 and 2).

Item	NC	NC + Coated ^B^	NC + Uncoated ^C^	NC + Combined ^D^	P_r_ > F ^E^	Pooled SEM ^E^
Body weight, g/bird						
Day 1	43.1	43.4	43.3	43.0	0.614	0.25
Day 14	451 ^b^	487 ^a^	488 ^a^	480 ^ab^	0.019	17.4
Day 21	909 ^b^	993 ^a^	995 ^a^	1002 ^a^	<0.001	27.2
Feed consumption, g/bird						
Day 1 to 14	524	548	548	543	0.225	18.0
Day 1 to 21	1151 ^b^	1231 ^a^	1206 ^a^	1216 ^a^	0.002	28.1
Mortality adjusted FCR, g:g						
Day 1 to 14	1.29 ^a^	1.24 ^b^	1.25 ^ab^	1.24 ^b^	0.016	0.029
Day 1 to 21	1.34 ^a^	1.30 ^ab^	1.28 ^b^	1.28 ^b^	0.009	0.033
Mortality, %						
Day 1 to 14	0.00	2.50	4.17	1.67	0.212	3.88
Day 1 to 21	0.83	2.50	5.00	2.50	0.400	4.82

^A^ Phytase unit. OptiPhos^®^ (Huvepharma Inc., Peachtree City, GA, USA) provided 1000 FTU/kg of phytase activity per kg of diet. ^B^ OptiPhos^®^ Plus (Huvepharma Inc., Peachtree City, GA, USA) provided 1000 FTU/kg of phytase activity per kg of diet. ^C^ OptiPhos^®^ and ^D^ OptiPhos^®^ Plus combined to provide 1000 FTU/kg of phytase activity per kg of diet. ^E^ P-value of F statistic; SEM = standard error of the mean. ^a,b^ Means in the same row with different superscript letters are significantly different (*p* < 0.05).

**Table 4:** Influence of negative control (NC), coated phytase supplementation (NC + 1000 FTU ^A^ coated phytase), uncoated phytase supplementation (NC + 1000 FTU uncoated phytase), and combined phytase supplementation (NC + 500 FTU coated + 500 FTU uncoated) on day 21 broiler tibia shear strength and tibia ash (pooled data from trials 1 and 2).

Item	NC	NC + Coated ^B^	NC + Uncoated ^C^	NC + Combined ^D^	P_r_ > F	Pooled SEM
Bone Wt., g	5.0 ^b^	5.4 ^a^	5.5 ^a^	5.6 ^a^	<0.001	0.10
Shear strength, peak 1 ^E^, N	246 ^b^	365 ^a^	365 ^a^	343 ^a^	<0.001	11.1
Shear strength, peak 2 ^F^, N	261 ^b^	410 ^a^	398 ^a^	384 ^a^	<0.001	11.5
Tibia ash, %	47.16 ^b^	52.38 ^a^	52.12 ^a^	52.59 ^a^	0.003	0.310

^A^ Phytase unit. ^B^ OptiPhos^®^ (Huvepharma Inc., Peachtree City, GA, USA) provided 1000 FTU/kg of phytase activity per kg of diet. ^C^ OptiPhos^®^ Plus (Huvepharma Inc., Peachtree City, GA, USA) provided 1000 FTU/kg of phytase activity per kg of diet. ^D^ OptiPhos^®^ and OptiPhos^®^ Plus combined to provide 1000 FTU/kg of phytase activity per kg of diet. ^E^ Force required to crack the bone. ^F^ Force required to break the bone. ^a,b^ Means in the same row with different superscript letters are significantly different (*p* < 0.05).

**Table 5:** Influence of negative control (NC), coated phytase supplementation (NC + 1000 FTU ^A^ coated phytase), uncoated phytase supplementation (NC + 1000 FTU uncoated phytase), and combined phytase supplementation (NC + 500 FTU coated + 500 FTU uncoated) on phytate phosphorus concentrations in feces from 21-day old broilers (pooled data from trials 1 and 2).

Item	NC	NC + Coated ^B^	NC + Uncoated ^C^	NC + Combined ^D^	P_r_ > F	Pooled SEM
Fecal phytate P, mg/kg	2.423 ^a^	630 ^b^	510 ^b^	552 ^b^	< 0.001	94.9

^A^ Phytase unit. ^B^ OptiPhos^®^ (Huvepharma Inc., Peachtree City, GA, USA) provided 1000 FTU/kg of phytase activity per kg of diet. ^C^ OptiPhos^®^ Plus (Huvepharma Inc., Peachtree City, GA, USA) provided 1000 FTU/kg of phytase activity per kg of diet. ^D^ OptiPhos^®^ and OptiPhos^®^ Plus combined to provide 1000 FTU/kg of phytase activity per kg of diet. ^a,b^ Means in the same row with different superscript letters are significantly different (*p* < 0.05).

## Data Availability

All data are contained within the article.

## References

[1] De Sousa J.P., Albino L.F., Vaz R., Rodrigues K.F., Da Silva G.F., Renno L.N., Barros V., Kaneko I.N. (2015). The effect of dietary phytase on broiler performance and digestive, bone, and blood biochemistry characteristics. Rev. Bras. Cienc. Avic..

[2] Selle P.H., Ravindran V. (2007). Microbial phytase in poultry nutrition. Anim. Feed Sci. Technol..

[3] Dersjant-Li Y., Awati A., Schulze H., Partridge G. (2015). Phytase in non-ruminant animal nutrition: A critical review on phytase activities in the gastrointestinal tract and influencing factors. J. Sci. Food Agric..

[4] Cowieson A.J., Acamovic T., Bedford M.R. (2004). The effects of phytase and phytic acid on the loss of endogenous amino acids and minerals from broiler chickens. Br. Poult. Sci..

[5] Woyengo T.A., Nyachoti C.M. (2011). Review: Supplementation of phytase and carbohydrases to diets for poultry. Can. J. Anim. Sci..

[6] Woyengo T.A., Nyachoti C.M. (2013). Review: Anti-nutritional effects of phytic acid in diets for pigs and poultry–current knowledge and directions for future research. Can. J. Anim. Sci..

[7] Walk C.L., Rama Rao S.V. (2020). Increasing dietary phytate has a significant anti-nutrient effect on apparent ileal amino acid digestibility and digestible amino acid intake requiring increasing doses of phytase as evidenced by prediction equations in broilers. Poult. Sci..

[8] Dersjant-Li Y., Davin R., Christensen T., Kwakernaak C. (2021). Effect of two phytases at two doses on performance and phytate degradation in broilers during 1-21 days of age. PLoS ONE.

[9] Broch J., dos Santos E.C., Damasceno J.L., Nesello P.D.O., de Souza C., Eyng C., Pesti G.M., Nunes R.V. (2020). Phytase and phytate interactions on broilers’ diet at 21 days of age. J. Appl. Poult. Res..

[10] Selle P.H., Cowieson A.J., Ravindran V. (2009). Consequences of calcium interactions with phytate and phytase for poultry and pigs. Livest. Sci..

[11] Cowieson A.J., Acamovic T., Bedford M.R. (2006). Phytic acid and phytase: Implications for protein utilization by poultry. Poult. Sci..

[12] Ayres V.E., Jackson M.E., Cantley S.A., Rochell S.J., Crumpacker C.D., Lee D.T., Bodle B.C., Pacheco W.J., Rueda M.S., Bailey C.A. (2021). Multiexperiment evaluation of increasing phytase activity from Optiphos^®^ and Optiphos Plus^®^ on 21-day broiler performance and tibia mineralization. J. Appl. Poult. Res..

[13] Yu S., Cowieson A., Gilbert C., Plumstead P., Dalsgaard S. (2012). Interactions of phytate and myo-inositol phosphate esters (IP1-5) including IP5 isomers with dietary protein and iron and inhibition of pepsin. J. Anim. Sci..

[14] Vats P., Banerjee U.C. (2004). Production studies and catalytic properties of phytases (myo-inositolhexakisphosphate phosphohydrolases): An overview. Enzym. Microb. Technol..

[15] Adeola O., Cowieson A.J. (2011). BOARD-INVITED REVIEW: Opportunities and challenges in using exogenous enzymes to improve nonruminant animal production. J. Anim. Sci..

[16] Singh P.K. (2008). Significance of phytic acid and supplemental phytase in chicken nutrition: A review. World’s Poult. Sci. J..

[17] Cowieson A.J., Acamovic T., Bedford M.R. (2006). Supplementation of corn-soy-based diets with an Eschericia coli-derived phytase: Effects on broiler chick performance and the digestibility of amino acids and metabolizability of minerals and energy. Poult. Sci..

[18] Walk C.L., Santos T.T., Bedford M.R. (2014). Influence of superdoses of a novel microbial phytase on growth performance, tibia ash, and gizzard phytate and inositol in young broilers. Poult. Sci..

[19] Olukosi O.A., Kong C., Fru-Nji F., Ajuwon K.M., Adeola O. (2013). Assessment of a bacterial 6-phytase in the diets of broiler chickens. Poult. Sci..

[20] Walk C.L., Bedford M.R., McElroy A.P. (2012). Influence of limestone and phytase on broiler performance, gastrointestinal pH, and apparent ileal nutrient digestibility. Poult. Sci..

[21] Ptak A., Bedford M.R., Świątkiewicz S., Żyła K., Józefiak D. (2015). Phytase modulates ileal microbiota and enhances growth performance of the broiler chickens. PLoS ONE.

[22] Campasino A., York T., Wyatt C., Bedford M.R., Dozier W.A. (2014). Effect of increasing supplemental phytase concentration in diets fed to Hubbard × Cobb 500 male broilers from 1 to 42 days of age. J. Appl. Poult. Res..

[23] Woyengo T.A., Slominski B.A., Jones R.O. (2010). Growth performance and nutrient utilization of broiler chickens fed diets supplemented with phytase alone or in combination with citric acid and multicarbohydrase. Poult. Sci..

[24] Wu D., Wu S.B., Choct M., Swick R.A. (2015). Comparison of 3 phytases on energy utilization of a nutritionally marginal wheat-soybean meal broiler diet. Poult. Sci..

[25] Sulabo R.C., Jones C.K., Tokach M.D., Goodband R.D., Dritz S.S., Campbell D.R., Ratliff B.W., DeRouchey J.M., Nelssen J.L. (2011). Factors affecting storage stability of various commercial phytase sources. J. Anim. Sci..

[26] Nováková J., Vértesi A., Béres E., Petkov S., Niederberger K.E., van Gaver D., Hirka G., Balázs Z. (2021). Safety assessment of a novel thermostable phytase. Toxicol. Rep..

[27] Cobb-Vantress, Inc. (2022). Cobb 500: Broiler Performance and Nutrition Supplement. https://www.cobb-vantress.com/assets/Cobb-Files/product-guides/5502e86566/2022-Cobb500-Broiler-Performance-Nutrition-Supplement.pdf.

[28] National Research Council (1994). Nutrient Requirements of Poultry.

[29] Downs K.M., Gulizia J.P., Stafford E.K., Pacheco W.J. (2022). Influence of varying dietary kudzu leaf meal particle size on performance, breast weight, and organ weight of broiler chickens from 1 to 21 days of age. Poultry.

[30] ASAS/ADSA/PSA (2020). Husbandry, housing, and biosecurity. Guide for the Care and Use of Agricultural Animal in Agricultural Research and Teaching.

[31] ASAS/ADSA/PSA (2020). Environmental enrichment. Guide for the Care and Use of Agricultural Animal in Agricultural Research and Teaching.

[32] American Veterinary Medical Association (2020). AVMA Guidelines for the Euthanasia of Animals.

[33] ASABE (2017). Shear and Three-Point Bending Test of Animal Bone.

[34] Hall L.E., Shirley R.B., Bakalli R.I., Aggrey S.E., Pesti G.M., Edwards H.M. (2003). Power of two methods for the estimation of bone ash of broilers. Poult. Sci..

[35] SAS Institute (2017). SAS/STAT® User’s Guide Version 14.3.

[36] Babatunde O.O., Cowieson A.J., Wilson J.W., Adeola O. (2019). Influence of age and duration of feeding low-phosphorus diet on phytase efficacy in broiler chickens during the starter phase. Poult. Sci..

[37] Babatunde O.O., Cowieson A.J., Wilson J.W., Adeola O. (2019). The impact of age and feeding length on phytase efficacy during the starter phase of broiler chickens. Poult. Sci..

[38] Batal A.B., Parsons C.M. (2002). Effects of Age on Nutrient Digestibility in Chicks fed Different Diets. Poult. Sci..

[39] Leyva-Jimenez H., Alsadwi A.M., Gardner K., Voltura E., Bailey C.A. (2019). Evaluation of high dietary phytase supplementation on performance, bone mineralization, and apparent ileal digestible energy of growing broilers. Poult. Sci..

[40] Kozlowski K., Nollet L., Lanckriet A., Vanderbeke E., Mielnik P., Outchkourov N., Petkov S. (2019). Effect of different phytases derived from E. coli AppA gene on the performance, bone mineralisation and nutrient digestibility of broiler chicken. J. Appl. Anim. Nutr..

[41] Sens R.F., Bassi L.S., Almeida L.M., Rosso D.F., Teixeira L.V., Maiorka A. (2021). Effect of different doses of phytase and protein content of soybean meal on growth performance, nutrient digestibility, and bone characteristics of broilers. Poult. Sci..

[42] Beeson L.A., Walk C.L., Bedford M.R., Olukosi O.A. (2017). Hydrolysis of phytate to its lower esters can influence the growth performance and nutrient utilization of broilers with regular or super doses of phytase. Poult. Sci..

[43] Kriseldi R., Walk C.L., Bedford M.R., Dozier W.A. (2021). Inositol and gradient phytase supplementation in broiler diets during a 6-week production period: 1. effects on growth performance and meat yield. Poult. Sci..

[44] Dos Santos T.T., Srinongkote S., Bedford M.R., Walk C.L. (2013). Effect of high phytase inclusion rates on performance of broilers fed diets not severely limited in available phosphorus. Asian-Aust. J. Anim. Sci..

[45] Dersjant-Li Y., Evans C., Kumar A. (2018). Effect of phytase dose and reduction in dietary calcium on performance, nutrient digestibility, bone ash and mineralization in broilers fed corn-soybean meal-based diets with reduced nutrient density. Anim. Feed Sci. Technol..

[46] Broch J., Nunes R.V., Eyng C., Pesti G.M., de Souza C., Sangalli G.G., Fascina V., Teixeira L. (2018). High levels of dietary phytase improves broiler performance. Anim. Feed Sci. Technol..

[47] Campbell G.L., Bedford M.R. (1992). Enzyme applications for monogastric feeds: A review. Can. J. Anim. Sci..

[48] Cardoso Júnior A., Rodrigues P.B., Bertechini A.G., de Freitas R.T.F., de Lima R.R., Lima G.F.R. (2010). Levels of available phosphorus and calcium for broilers from 8 to 35 days of age fed rations containing phytase. R. Bras. Zootec..

[49] Pereira R., Menten J., Romano G.G., Silva C., Zavarize K.C., Barbosa N. (2012). Efficiency of a bacterial phytase to release phytate phosphorus in broiler chicken diets. Arq. Bras. Med. Vet. Zootec..

[50] Bougouin A., Appuhamy J.A.D.R.N., Kebreab E., Dijkstra J., Kwakkel R.P., France J. (2014). Effects of phytase supplementation on phosphorus retention in broilers and layers: A meta-analysis. Poult. Sci..

[51] Chung T.K., Rutherfurd S.M., Thomas D.V., Moughan P.J. (2013). Effect of two microbial phytases on mineral availability and retention and bone mineral density in low-phosphorus diets for broilers. Br. Poult. Sci..

[52] Babatunde O.O., Bello A., Dersjant-Li Y., Adeola O. (2022). Evaluation of the responses of broiler chickens to varying concentrations of phytate phosphorus and phytase. II. Grower phase (day 12-23 post hatching). Poult. Sci..

[53] Martins B.A.B., Borgatti L.M.D.O., Souza L.W.D.O., Robassini S.L.D.A., de Albuquerque R. (2013). Bioavailability and poultry fecal excretion of phosphorus from soybean-based diets supplemented with phytase. R. Bras. Zootec..

[54] Kriseldi R., Johnson J.A., Walk C.L., Bedford M.R., Dozier W.A. (2021). Influence of exogenous phytase supplementation on phytate degradation, plasma inositol, alkaline phosphatase, and glucose concentrations of broilers at 28 days of age. Poult. Sci..

[55] Gautier A.E., Walk C.L., Dilger R.N. (2018). Effects of a high level of phytase on broiler performance, bone ash, phosphorus utilization, and phytate dephosphorylation to inositol. Poult. Sci..

[56] Kriseldi R., Walk C.L., Bedford M.R., Dozier W.A. (2021). Inositol and gradient phytase supplementation in broiler diets during a 6-week production period: 2. Effects on phytate degradation and inositol liberation in gizzard and ileal digesta contents. Poult. Sci..

[57] Abbasi F., Fakhur-un-Nisa T., Liu J., Luo X., Abbasi I.H.R. (2019). Low digestibility of phytate phosphorus, their impacts on the environment, and phytase opportunity in the poultry industry. Environ. Sci. Pollut. Res..

